# Case of Protracted and Complicated Disease

**Published:** 1871-09

**Authors:** L. D. Robinson

**Affiliations:** Brookfield, Mo.


### CASE OF PROTRACTED AND COMPLICATED DISEASE.

By L. D. ROBINSON, M.D., of Brookfield, Mo.

Having had, by the kindness and professional courtesy of my esteemed friend Dr. R. J. Scott, a limited connection with a case of rather unusual interest, in many respects, I propose to furnish you the facts for the pages of the *Examiner*, as they have been given me by one brief interview with the patient, by Dr. Scott, and as revealed by the autopsy.

Mrs. Logsdon, aged 40 years, of nervo-sanguine temperament, said, that while a resident in the State of Illinois, some seventeen years ago, she was attacked with what *she* believed to be milk-sickness—but her medical attendant termed it malarial fever; that the attack was severe and of long continuance; that she dreamed, while in a state of semi-delirium, that if she would swallow a couple of sewing needles she would soon fully recover; that she was a seamstress, had an ivory needle case nearly full of assorted needles, with top screwed on, that her best impressions were that she swallowed it; at all events, she could never, after her recovery, find the needle case. This is the history of the matter given by the patient, both to Dr. Scott, her medical adviser, and myself.

Some three years since, she lost by death her husband, from the effects of which she seemed to never fully recover, and in August of 1869, removed to this vicinity, and almost immediately called upon Dr. Scott for treatment. The Dr. says he found her considerably emaciated, and her general health much under par, with an obstinate vomiting; but discerned nothing unusual in her case. He diagnosed chronic inflammation of stomach, and instituted treatment accordingly—keeping it up from month to month with varied, and, upon the whole, rather unsatisfactory results, up to the month of April, 1870; the patient emitting pus from the stomach, and, at rare times, per anum.

Finally, during the month of April, 1870, Dr. Scott was summoned hastily to the couch of his patient, and she described a peculiar stinging, painful sensation in the epigastric region when she moved her body, and upon examination, the Dr. discovered, immediately underneath the skin, evidences of some foreign substance, and upon cutting down upon it discovered and removed two needles. It was then the lady first told him the needle-swallowing story. I should have stated, that the needles found in the epigastric region, were bright, free from rust, and points keen and sharp!

The Dr. continued the treatment of the case, endeavoring to tone up the general health, and gastric mucous membrane, but with varied, and only partial, success—meantime he continued to extract needles, as they would come to or near the cutaneous surface, from one to eleven at an operation, the greater number from the epigastric and hypochondriac regions, removing a few, however, from the upper and lower extremities—numbering in all, including pieces, *one hundred and twenty-one needles*, all of which he still has in his possession!

Those removed from the lower extremities and feet, were somewhat corroded or rusted, the points being considerably dulled or blunted by the action on them of the acids contained in the juices of the body.

For one month prior to her demise she seemed to be in rather a better state of general health than usual, and did quite an amount of ordinary housework daily, as was her custom during the entire time Dr. Scott had her case under treatment, with the exception of an attack of prostration confining her to bed for a period of near seven weeks, which occurred something over one year ago. She had the care of three children.

On Thursday, the 20th July, 1871, she did a moderate washing in the forenoon, cooked the dinner meal for children and self, eating heartily, after which began preparation for ironing, meantime commenced mopping house-floor in a stooping posture. Upon raising up rather suddenly, was instantaneously attacked by syncope, and sank upon the floor; from that she did not react, but remained unconscious, with lividity of cutaneous surface, and general visceral congestions, until death closed the scene within two hours from the sudden attack.

When I saw her, and conversed with her, on or about the 1st of July, and heard her history of herself, her sickness, the swallowing of needle-case, &c., &c., and the loss, by disease and death of her husband, some three years ago, my opinion was, and still is, that her bad general health, the cares and duties of life, in bearing and rearing a tolerably large family of children, and finally, the loss of her husband, had induced chronic or habitual nervous debility, and, as a consequence, chronic hysteria, and that, in its turn, finally brought about that inexplicable mania which so often pertains to such cases; that instead of having swallowed, *seventeen years ago*, an ivory needle-case, containing needles, she had been swallowing them, from time to time, for the past two years, in order to become notorious, and secure the sympathy of her children, friends, and medical advisers; and Dr. Scott, with his very intimate intercourse with, and knowledge of, the case fully agrees with me in this view of the case.

*Item.* She had at one time strenuously claimed that she went *seven weeks* without one particle of nourishment, (other than a small amount of coffee daily,) and without a single evacuation of the bowels for five weeks of the seven! Yet was not in the least emaciated more than usual!

Also, a few days prior to death, she seemed to think the Dr. was not taking as much interest in her case as usual, as no needles had been extracted for several weeks, and but few, if any, persons had called to see her, as many had hitherto been doing, the people regarding her a sort of prodigy. She sent to the Dr., in a paper, what she represented as a portion of the ivory case, which she alleged, had been ejected from the stomach by emesis a few hours previously. Upon careful examination, the specimen sent by her, was found to be, unmistakably, pulverized muscle shell!

Autopsy.—Owing to a combination of circumstances, the *post mortem* examination was not had until forty-eight hours after death. In company with, and through the kindness and courtesy of, Dr. Scott, he and myself conducted the autopsy by first removing and examining the stomach, which we found entirely empty, but inflated with gas. There was general softening of its mucous membrane, with general dark-grayish discoloration.

We next examined the liver, spleen, pancreas, and abdominal viscera in general, finding nothing unusual or abnormal, save a pale, flabby, attenuated condition of the parenchyma of liver, spleen, pancreas, &c.

Upon opening the thorax, we first observed the heart to be about two and a-half inches above its normal position; whether this abnormality was congenital or otherwise could not be determined. The heart was much attenuated in its walls, and also atrophied—the walls of the auricles being almost as thin as the paper I write on. The walls of the ventricles correspondingly so. When it had been removed from the thorax and was laid upon the table, instead of maintaining its normal shape and rotundity, it would, of its own weight, flatten down into a shapeless mass, like a wet cloth.

The lungs were thickly studded with the miliary tubercle, but no vomica. The patient had never manifested any symptoms of lung trouble. Right lung and pulmonary pleura were firmly adherent to costal pleura; about two inches in diameter of the superior lobe of lung being thus adherent to walls of the chest.

We then examined the œsophagus, along its entire length, in order to ascertain if any recently-swallowed needles had lodged there, but found none.

The patient was very anæmic, and blood much impoverished.

Now, what caused the sudden death, and what was the mode of dying? Had the needles anything to do in the case? Dr. Scott arrived at the bedside of the patient near one hour before death, and from the Dr.'s description of the case, and the death, and from what light was thrown upon the subject by the hurried autopsy, we have come to the conclusion that it was death by syncope.

The heart being atrophied, and very weak, the blood very deficient in iron, especially the bloodvessels being much relaxed, and their calibre enlarged, when the patient stooped over, there was an unnaturally full rush of blood to the brain, and when the erect posture was assumed, there was an equally free and abnormal efflux of blood from the vessels of the brain, and a fatal syncope the result—the enfeebled heart and arteries, and want of natural stimulant property in the blood to prompt the heart to action, being sufficient evidence of the fact to sustain this hypothesis.

Again:—Were the needles swallowed seventeen years ago, screwed up tightly in any ivory case, as the patient claimed? Or is our prediction correct in taking the position that she had been swallowing them, from time to time, within the past two or three years?

P.S.—I am indebted to Dr. R. J. Scott, of this place, for all the knowledge I have of this interesting case, and it is by his request that I have thus shaped up the facts for publication.

